# Development of Soy-Based Meat Analogues via Wet Twin-Screw Extrusion: Enhancing Textural and Structural Properties Through Whole Yeast Powder Supplementation

**DOI:** 10.3390/foods14142479

**Published:** 2025-07-15

**Authors:** Shikang Tang, Yidian Li, Xuejiao Wang, Linyan Zhou, Zhijia Liu, Lianzhou Jiang, Chaofan Guo, Junjie Yi

**Affiliations:** 1 Faculty of Food Science and Engineering, Kunming University of Science and Technology, Kunming 650500, China; 15969040899@163.com (S.T.); liyidian0109@163.com (Y.L.); wangxuejiao173@hotmail.com (X.W.); zhoulinyan916@hotmail.com (L.Z.); zhijia_liu@outlook.com (Z.L.); junjieyi@kust.edu.cn (J.Y.); 2 Yunnan Key Laboratory of Plateau Food Advanced Manufacturing, Kunming 650500, China; 3 Yunnan International Joint Laboratory of Green Food Processing, Kunming 650500, China; 4 International Green Food Processing Research and Development Center of Kunming City, Kunming 650500, China; 5 College of Food Science, Northeast Agricultural University, Harbin 150030, China; jlzname@163.com

**Keywords:** whole yeast powder, soya protein, high-moisture extrusion, artificial meat, textural properties

## Abstract

Amid growing global concerns about environmental sustainability and food security, plant-based meat substitutes have emerged as a promising alternative to conventional meat. However, current formulations, especially those based on soy protein isolate (SPI) often fail to replicate the desired texture and structural integrity. To address this limitation, this study aimed to evaluate the use of whole yeast powder (WYP) combined with SPI for producing plant-based meat analogues via high-moisture extrusion. Seven groups were designed: a control group with 0% WYP, five treatment groups with 5%, 10%, 20%, 30%, and 40% WYP, and one reference group containing 20% yeast protein powder (YPP). Although lower in protein content than yeast protein powder (YPP), whole yeast powder exhibits superior water-binding capacity and network-forming ability owing to its complex matrix and fiber content. At a 20% inclusion level, whole yeast powder demonstrated a higher fibrous degree (1.84 ± 0.02 vs. 1.81 ± 0.04), greater hardness (574.93 ± 5.84 N vs. 531.18 ± 17.34 N), and increased disulfide bonding (95.33 ± 0.92 mg/mL vs. 78.41 ± 0.78 mg/mL) compared to 20% YPP. Scanning electron microscopy (SEM) and low-field nuclear magnetic resonance (LF-NMR) revealed that whole yeast powder facilitated the formation of aligned fibrous networks and enhanced water binding. Fourier transform infrared spectroscopy (FTIR) confirmed an increase in β-sheet content (0.267 ± 0.003 vs. 0.260 ± 0.003), which contributed to improved protein aggregation. Increasing the WYP content to 30–40% led to a decline in these parameters, including a reduced fibrous degree (1.69 ± 0.06 at 40% WYP) and weakened molecular interactions (*p* < 0.05). The findings highlight 20% WYP as the optimal substitution level, offering superior textural enhancement and fibrous structure formation compared to YPP. These results suggest that WYP is not only a cost-effective and processing-friendly alternative to YPP but also holds great promise for scalable industrial application in the plant-based meat sector. Its compatibility with extrusion processes and ability to improve sensory and structural attributes supports its relevance for sustainable meat analogue production.

## 1. Introduction

The global demand for meat products has steadily increased, driven by factors such as population growth, rapid urbanization, industrial expansion, and rising household incomes [1]. Consequently, this surge in demand has intensified environmental challenges, including excessive land use, water scarcity, increased pressure to control animal diseases, and rising greenhouse gas emissions [2]. Plant-based meat analogues, designed to replace conventional meat, offer considerable environmental benefits and are emerging as a promising strategy to alleviate the ecological burden of the meat industry. Driven by growing sustainability awareness, ethical concerns over animal consumption, and increasing health-conscious diets, plant-based meat alternatives are rapidly expanding across diverse consumption contexts and accelerating the growth of the alternative protein market [3]. Achieving realistic texture, authentic flavor, and nutritional equivalence is a major technical barrier in this field [2]. Currently, the main methods for producing vegetable-based meat analogues involve high-moisture extrusion (HME) technology and food 3D printing [4,5]. HME, defined by water content exceeding 40%, offers advantages including lower energy consumption, reduced waste, and improved operational efficiency. As a result, HME has become the leading technology for plant-based meat analogue production [6].

Currently, artificial meat is primarily composed of plant proteins such as soy protein, wheat gluten, and pea protein [7,8], with soy protein isolate (SPI) being the most widely used due to its functionality and availability. However, SPI-based products still exhibit limitations, including environmental concerns related to soybean cultivation and challenges in achieving optimal texture and flavor [9]. Although recent developments in SPI-based analogues have advanced significantly, achieving meat-like structural performance remains difficult.

Most prior research has focused on yeast protein powder (YPP), a purified extract derived from fermented yeast, and its mixture with SPI for use in plant-based meat analogues. One study showed that extrusion of YPP and SPI at a ratio of 40 g/100 g yielded the best structural performance, including (*p* < 0.05) high hardness (523.94 ± 11.91 N), chewiness (724.55 ± 22.89 N), and fibrous degree (2.06 ± 0.15) [10]. However, research on whole yeast powder (WYP) is limited. WYP contains 40–60% crude protein on a dry matter basis and retains a broader nutritional profile, including carbohydrates, crude fat, and dietary fiber [11]. Unlike YPP, which requires extraction and purification, WYP is produced with minimal processing and has shown better cost-effectiveness and processing adaptability in food applications. While WYP has been applied in products such as ice cream and beverages, and shown benefits such as gut microbiota-related anti-aging effects [4], it is still primarily used in animal feed [12].

Despite its favorable composition, WYP’s potential in high-moisture extrusion has been largely overlooked. Current studies focus almost exclusively on YPP [6], leaving a gap in understanding how WYP behaves under high-moisture, high-shear processing. To date, no studies have specifically investigated whole yeast powder, which is the core reason for selecting WYP as an alternative protein material in this study. Therefore, this study aimed to evaluate the potential of whole yeast powder as a cost-effective and functional additive to SPI in high-moisture extruded meat analogues. Through comparative analysis with YPP, this study seeks to clarify the effects of WYP on extrudability, fiber formation, and texture enhancement, and to provide a basis for its application in industrial-scale formulation development.

This study produced plant-based meat analogues using high-moisture extrusion with varying ratios of whole yeast powder and soy protein isolate (SPI), using 20% yeast protein powder (YPP) as the reference. The physicochemical and structural properties were evaluated, including color measurements, scanning electron microscopy (SEM), surface analysis, texture profiling, water distribution (via LF-NMR), protein secondary structure, intermolecular interactions, surface hydrophobicity, and Fourier transform infrared (FTIR) spectroscopy. Comparative analysis between 20% YPP and varying WYP substitution levels revealed the underlying mechanisms of fibrous structure formation in plant-based meat, providing a scientific basis for the application of whole yeast powder in industrial-scale meat analogue production.

## 2. Materials and Methods

### 2.1. Raw Materials and Sample Preparation

For this study, SPI was sourced from Linyi Shansong Biological Products Co., Ltd. (Linyi, China); the protein sample contained 91.4 g/100 g of protein and 6.56 g/100 g of moisture on a dry basis. The whole yeast powder (dry-based), provided by Angel Yeast Co., Ltd. (Yichang, China), comprised 48.3 g/100 g of protein, 13.1 g/100 g of carbohydrates, 4.7 g/100 g of crude fat, and 23.53 g/100 g of crude dietary fiber. The yeast protein powder content was measured to be 84.2 g/100 g on a dry basis, with a moisture content of 3.3 g/100 g. Sodium chloride, urea, phosphate buffer solution, and β-mercaptoethanol were all purchased from Kunming Meibo Biotechnology Co., Ltd. (Kunming, China). The reagents required for the experiments were of analytical grade. To prepare the mixtures, different proportions of whole yeast powder and SPI were combined, with the following yeast powder-to-SPI ratios: 0.5:9.5, 1:9, 2:8, 3:7, and 4:6. Table 1 provides the precise compositions and sample quantities.

### 2.2. HME Process

All high-moisture extrusion experiments were conducted using a laboratory-scale twin-screw extruder (FMHE22; Fumai Food Co., Ltd., Linyi, China). The feed rate of the SPI–WYP dry blend was maintained at 9.5 kg/h, with deionized water introduced at 5.9 kg/h. The extruder was equipped with seven sequential heating zones, with temperatures progressively set at 60, 80, 90, 120, 130, 140, and 140 °C. A schematic diagram of the twin-screw extruder is presented in the Supplementary Material. For each formulation, raw materials were divided into three portions and processed under identical operating conditions, with each sample prepared in triplicate. The resulting extrudates were subsequently divided into two portions: one was vacuum freeze-dried, ground, and sieved through a 100-mesh screen for further analysis; the other was stored at 4 °C for subsequent use.

### 2.3. Microstructure and Visual

The samples were observed using a scanning electron microscope (Apreo 2C, Thermo Fisher Scientific, Bremen, Germany). The observation process and parameters followed the method described in [13]; the operating voltage of the SEM was 10 kV. Magnifications of ×500, ×1500, were used.

The samples were cut into dimensions of 40 mm × 20 mm × 10 mm using a knife and were fixed onto an iron rod for macrostructure observation through photography.

To further evaluate the alignment and density of fibrous structures, fiber orientation analysis was performed using ImageJ v.1.54 and its OrientationJ plugin. The fiber alignment of the extrudate microstructure was quantitatively assessed using the OrientationJ plugin in ImageJ (National Institutes of Health, Bethesda, MD, USA). SEM images (×1500 magnification) were first converted to 8-bit grayscale and then analyzed using OrientationJ Distribution to extract the angular distribution of fiber orientation. The analysis parameters included a smoothing factor of 2 and a region of interest (ROI) covering the central textured area. The output included an orientation histogram ranging from −90° to +90°, with the peak angle representing the dominant fiber alignment direction. A sharper and higher peak near 0° indicated better unidirectional alignment. The coherency index and standard deviation were also extracted to evaluate the degree of orientation quantitatively. SEM images of samples with varying substitution levels of whole yeast powder or yeast protein powder were also processed using ImageJ binary thresholding. Additional details are provided in the Supplementary Material (Figure S4).

The extrudates were torn along the direction of mold extrusion, fixed onto an iron rod, and photographed in a studio using a smartphone (iPhone 15, Apple Co., Cupertino, CA, USA) [14].

### 2.4. Textural

The texture characteristics of the samples were tested using a texture analyzer (TA-XT Plus, Stable Micro Systems, Godalming, UK). The samples were cut into strips of 20 mm × 20 mm × 10 mm using a knife and fixed onto the testing platform. The testing parameters followed the method described in [15]. The vertical force (FV) and parallel force (FL) were recorded, and their ratio (FV/FL) was used to evaluate the degree of fiber alignment (fiberization). In addition, chewiness and hardness were measured to characterize the overall texture properties of the samples.

### 2.5. LF-NMR Determination

Low-field nuclear magnetic resonance (LF-NMR) measurements were performed using a benchtop NMR analyzer (PQ001-20-020V, Niumag Analytical Instrument Co., Ltd., Suzhou, China) operating at 20 MHz. The Carr–Purcell–Meiboom–Gill (CPMG) pulse sequence was used to determine the spin–spin relaxation time (T_2_) and the corresponding relative peak area (P_2_). The acquisition parameters were set as follows: echo time (TE) of 0.3 ms, 1024 echoes per scan, repetition time (TR) of 5000 ms, and four accumulated scans. Signal acquisition and T_2_ distribution analysis were performed using the MultiExp Inv 2023 software (Niumag).

### 2.6. Intermolecular Interaction Forces

The intermolecular interactions of proteins were tested with slight modifications based on the method described in [16]. The samples were dissolved separately in the following solutions: 0.05 mol/L NaCl (S1), 0.6 mol/L NaCl (S2), 0.6 mol/L NaCl + 1.5 mol/L urea (S3), 0.6 mol/L NaCl + 8 mol/L urea (S4), and 0.6 mol/L NaCl + 8 mol/L urea + 0.5 mol/L β-mercaptoethanol (S5). The supernatant was collected, and the protein concentration was determined using a BCA protein assay kit. The differences in protein concentration between S5 and S4, S4 and S3, S3 and S2, and S2 and S1 were used to evaluate the contributions of ionic bonds, hydrogen bonds, hydrophobic interactions, and disulfide bonds, respectively.

### 2.7. Surface Hydrophobicity

The surface hydrophobicity of the sample was determined using the bromophenol blue (BPB) binding method, as referenced by [17]. The measurement parameters were set as follows: excitation wavelength 390 nm, emission wavelength 470 nm, slit width 5 nm, and scan speed 600 nm/min. 2 g of the sample was placed in 10 times the volume of PBS buffer (20 mmol/L, pH 6) and homogenized using a high-speed disperser at 18,000 rpm. The protein concentration in the suspension was then measured and adjusted to 5 mg/mL. Additionally, 1 mL of the sample suspension and PBS (control) were mixed with 200 μL of BPB (1 mg/mL) by vortexing. After stirring at room temperature for a period of time, all samples and the control were centrifuged at 4 °C, 2000× *g* for 15 min. The supernatant was collected, diluted 10 times with PBS, and the absorbance was measured at 595 nm. The amount of BPB bound was calculated as follows (1):

(1) $$\mathrm{Amount}\;\mathrm{of}\;\mathrm{BPB}\;\mathrm{bound}\;(\mu g)=200\;\mu g\;\times \;\frac{A_{C}-A_{S}}{A_{C}}$$

In the formula, *Ac* and *As* represent the absorbance values of the control and the sample at 595 nm, respectively.

### 2.8. Color Measurement

The color changes of the extrudates were measured using a colorimeter (Hunter Associate Lab, Reston, VA, USA). Samples were cut into blocks with dimensions of 20 × 20 × 10 mm, and the measurement parameters followed the procedure described in [18]. In the CIELAB color space, L* represents lightness, ranging from 0 (black) to 100 (white); a* and b* represent chromaticity, ranging from −128 to 127, where positive a* indicates red and negative a* indicates green, while positive b* indicates yellow and negative b* indicates blue. The total color difference (ΔE*) was calculated using the following Equation (2):

(2) $$\Delta E=\sqrt{(L_{1}^{*}-L_{C}^{*}{)}^{2}+(a_{1}^{*}-a_{c}^{*}{)}^{2}+(b_{1}^{*}-b_{c}^{*}{)}^{2}}$$

### 2.9. FTIR Spectroscopy Analysis

Changes in the protein secondary structure of the extruded samples were analyzed using Fourier transform infrared (FTIR) spectroscopy (Nicolet iS50; Thermo Fisher Scientific, Germany). The samples were mixed with potassium bromide (KBr) at a ratio of 1:100, and the spectra were recorded over the range of 500–4000 cm^−1^. The amide I region (1600–1700 cm^−1^) was used for secondary structure analysis, and peak fitting was conducted using PeakFit v4.12 software. Fourier self-deconvolution was applied to the spectra of extrudates containing different substitution levels of whole yeast powder (see Supplementary Figure S3).

### 2.10. Statistical Analysis

All results were obtained from three independent experiments and expressed as mean ± standard deviation (SD). Statistical analysis was performed using analysis of variance (ANOVA) in SPSS Statistics 27.0 software (IBM Corp., Armonk, NY, USA), followed by Tukey’s honestly significant difference (HSD) test for multiple comparisons. A significance level of *p* < 0.05 was considered statistically significant. Data visualization was performed using Origin 2021, and the types of graphs created include line charts and bar charts.

## 3. Results

### 3.1. Color Analysis

Color is one of the core attributes influencing product attractiveness and value, shaping the product’s visual identity while also implicitly reflecting its nutritional quality and market positioning [19].

As shown in Table 2, the brightness (L* value) increased progressively from 37.89 ± 0.23 at 0% WYP to 49.16 ± 0.70 at the highest substitution level (40% WYP). This suggests that the addition of whole yeast powder and yeast protein powder contributed to a brighter appearance, likely due to their inherent color properties. For the a* value, only minor variations were observed among samples, ranging from 3.17 ± 0.14 to 3.91 ± 0.04, indicating that substitution had no significant effect on the red–green color axis. However, the b* value increased from 14.86 ± 0.45 at 0% WYP to 18.22 ± 0.02 at 40% WYP, indicating enhanced yellowness with increasing substitution levels. This result is likely attributed to the intrinsic yellowness of whole yeast and yeast protein powders, which impart a more yellow hue to the product. Moreover, the total color difference (Δ*E*) increased from 0.00 at 0% substitution to 11.80 ± 0.24 at 40% WYP, suggesting that the overall color difference became more pronounced with increasing substitution, particularly at higher levels. Previous studies have shown that color changes in plant-based meat analogues are closely related to Maillard reactions occurring within the extruder barrel during processing [20,21].

The addition of whole yeast powder and yeast protein powder increased the brightness (L* value) and enhanced the yellowness (b* value) of the product, while having minimal impact on the red–green contrast (a* value). The total color difference (Δ*E*) also significantly increased with higher substitution levels, particularly at higher levels (e.g., 40% YP), reaching perceptible differences (Δ*E* > 3).

### 3.2. Microstructure and Visual Analysis

Scanning electron microscopy (SEM) enables the detailed visualization of the fibrous structure and internal microarchitecture of high-moisture mixed-protein extrudates [13,22].

**Figure 1:**
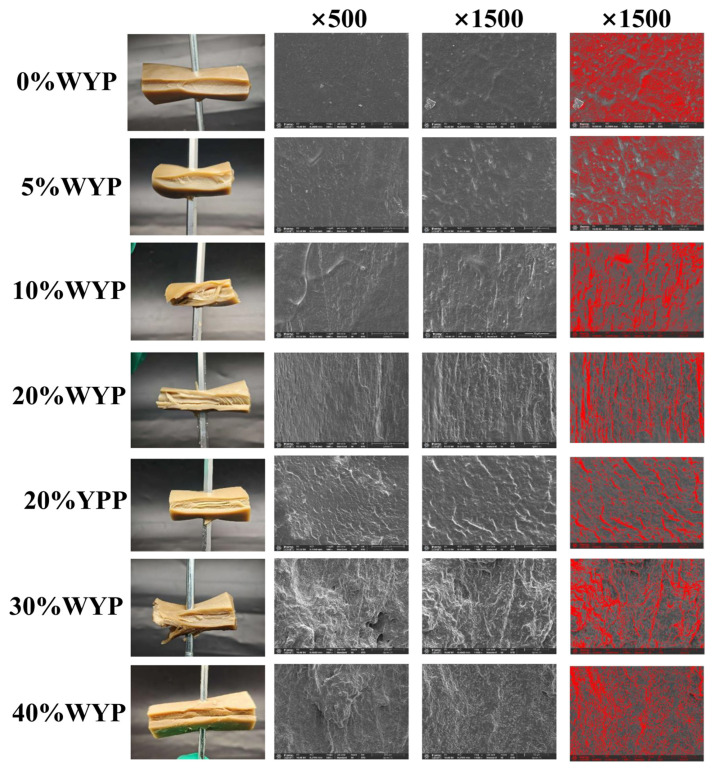
Comparison of the macroscopic appearance of high-moisture mixed-protein extrudates at different substitution levels and their SEM images at ×500, ×1500.The fourth column represents the binarized SEM image (×1500) of the extruded sample.

As shown in Figure 1, SEM images revealed that high-moisture extrudates composed solely of soy protein isolate (SPI) exhibited limited fibrous and interwoven structures in the absence of whole yeast powder (WYP) substitution [15]. When WYP substitution ranged from 5% to 20%, the formation of torn fibrous and interwoven structures progressively increased with rising WYP content. However, further substitution beyond 20% resulted in a decline: extrudates with 30% WYP showed fewer fibrous structures than those with 20%, and this reduction was more pronounced at 40% WYP. As shown in the binarized images (Figure 1), red-highlighted regions represent aligned microstructures with high edge responses, indicating the degree of fiber orientation and structural continuity. The control group (0% WYP) displayed scattered and irregular red areas, suggesting the absence of organized directional structures. As the WYP substitution level increased, particularly at 10% and 20%, the red regions became more continuous and vertically aligned, indicating the progressive development of fibrous protein networks. Notably, the 20% WYP sample exhibited the most prominent and organized vertical structures, confirming the highest degree of fiber alignment and structural organization. In contrast, the 20%YPP, 30%WYP, and 40%WYP groups showed disrupted and randomly oriented structures, with fragmented or curved patterns, indicating reduced fiber formation ability and less structural coherence. These results were validated through the texture tests below.

These findings indicated that whole yeast powder substitution between 5% and 20% increased fibrous interwoven structures in high-moisture mixed-protein extrudates, with the highest amount at 20%WYP. Adding more whole yeast powder beyond 20% disrupted the existing fibrous interwoven structure. Although 20%YPP extrudates showed some fibrous structure in both SEM images and visual observation [10], the 20%WYP extrudates exhibited more fibrous interwoven structures upon tearing, suggesting superior extrudability of 20%WYP over 20%YPP. A possible reason for this phenomenon is that under high pressure and shear forces in the twin-screw extruder, pure yeast protein powder undergoes denaturation, destroying its structure [23]. In contrast, whole yeast powder contains dietary fiber that does not denature, allowing more fibrous structures to remain in the mixed-protein extrudate. Similarly, ref. [14] reported that the incorporation of insoluble dietary fiber into soybean isolate protein during high-moisture extrusion promoted the formation of filamentous structures. In addition, the arrangement of these fiber structures follows the extrusion direction, which is similar to the experimental results reported by [24,25].

### 3.3. Texture Analysis

Texture is a critical characteristic of meat analogs, as it greatly influences their perceived mouthfeel and overall palatability. In this study, a texture analyzer was used to evaluate the impact of high-moisture extrusion parameters on the hardness, chewiness, and fibrousness of co-precipitated proteins. The degree of fiber formation was considered a key indicator of the fiber structure [15,22]. Numerous studies have indicated that the development of fibrous structures is strongly associated with protein–protein interactions during the extrusion process, particularly due to the oriented rearrangement of protein macromolecules [26,27].

As shown in Table 3, different whole yeast powder substitution levels exerted a pronounced effect on the products’ hardness and chewiness. As the WYP substitution ratio increased, both hardness and chewiness (measured in parallel and perpendicular directions) increased significantly, indicating a positive correlation between substitution level and textural properties. These findings are consistent with those reported by [10], in which yeast protein powder and soy protein were used in high-moisture extrusion.

According to Table 3, all samples exhibited a fibrous degree greater than 1, indicating the presence of fibrous structures in each sample [7]. Under low-to-moderate substitution ratios (5–20%WYP), the fibrous degree of the high-moisture mixed-protein extrudates increased with higher substitution levels, reaching a maximum (1.84 ± 0.02, *p* < 0.05) at 20%WYP. As the substitution level increased further (30–40%), it led to a considerable reduction in the fibrous degree, with the lowest value (1.69 ± 0.06, *p* < 0.05) observed at 40%WYP. This finding showed that an excessive amount of whole yeast powder could hinder the formation of fibrous structures [7]. A possible explanation is that the superior viscoelastic properties of the SPI–WYP and SPI–YPP mixtures may prolong their residence time within the extruder, the extended exposure to high shear forces and elevated temperatures promotes more extensive protein unfolding, thereby facilitating the development of stronger and more defined fibrous structures [28]. In most studies, the fibrous level of soybean protein-based meat analogs typically ranges from 0.7 to 1.7 [29]. However, in this study, when the whole yeast powder substitution level reached 20%, the fibrous level exceeded 1.7, reaching 1.84 ± 0.02. Moreover, the fibrous degree for whole yeast powder was higher than that for YPP (1.81 ± 0.04). Although there was no significant difference in fibrous degree between whole yeast powder and yeast protein powder, the extrudates containing whole yeast powder showed greater hardness and chewiness than those containing yeast protein powder. The observed difference may result from the dietary fiber content in whole yeast powder, whereas yeast protein powder is composed predominantly of purified protein and lacks fiber. Additionally, SEM images indicated that the fibrous structures formed by whole yeast powder were more orderly and complete than those formed by yeast protein powder, displaying a smoother appearance in the parallel direction. This finding suggests that 20%WYP achieved better extrudability than 20%YPP, indicating that whole yeast powder substitution promoted the formation of fibrous structures in meat analogs.

### 3.4. Low-Field NMR Analysis

Low-field nuclear magnetic resonance (LF-NMR) is widely employed to assess the distribution and physical state of water in food systems. The T_2_ relaxation time reflects the mobility of water molecules, typically ranging from 1 to 10 ms for bound water, 10 to 100 ms for immobilized water, and 100 to 1000 ms for free water [15]. A longer T_2_ relaxation time indicates greater water mobility, whereas a shorter T_2_ suggests restricted molecular movement [30,31]. Similarly, longer T_2_ values are associated with higher water activity, while shorter values indicate lower water activity levels [32].

**Figure 2:**
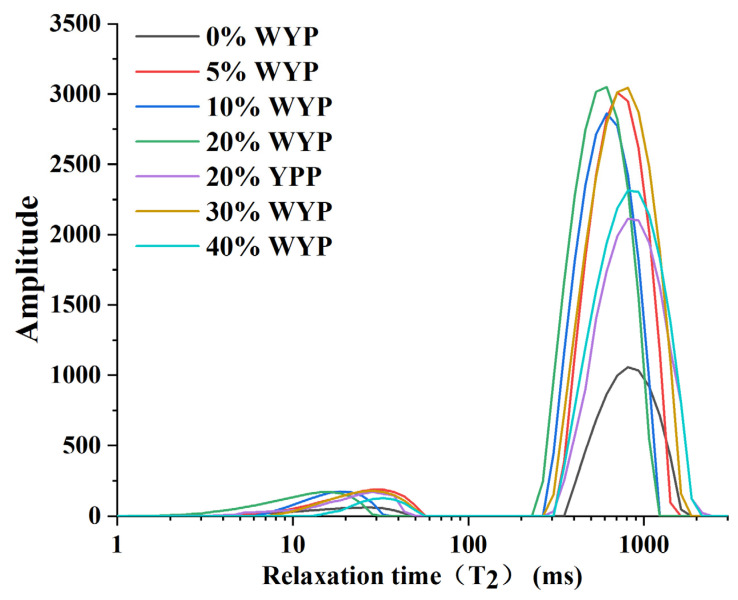
Water relaxation time curves of meat analogs at different substitution levels.

As shown in Figure 2, the T_2_ relaxation time of immobilized water significantly decreased at low-to-moderate WYP substitution levels (5–20%), suggesting enhanced water entrapment within the protein matrix and stronger interactions between the protein network and water molecules [33]. These results indicate that moderate WYP incorporation effectively enhances water-holding capacity by stabilizing water in a semi-bound state. However, when the substitution level exceeded 20%, the T_2_ relaxation time of immobilized water increased, indicating a reduced ability of the protein matrix to retain water, possibly due to structural disruption or excessive phase separation at higher WYP levels [33]. In comparison, the T_2_ relaxation time of bound water showed a slight decrease at lower substitution levels, followed by a moderate increase beyond 20%WYP, although the changes were less significant than those observed for immobilized water. In addition, the 0%WYP sample exhibited a very weak capacity to bind water molecules. T_2_ decreased as the whole yeast powder content increased from 5% to 20%, which may suggest a partial transition from immobilized to bound water and more intimate interactions between water and protein molecules. The reason for this phenomenon may be that the incorporation of whole yeast powder leads to a more compact network structure. Ref. [34] reached similar conclusions in their study of lemon juice gels used as 3D printing food materials.

Moreover, the T_2_ relaxation times of free water, immobilized water, and bound water in the 20%WYP extrudate were shorter than those in the 20%YPP extrudate, indicating a stronger water-binding capacity in the sample [33]. A higher degree of water binding typically reflects a denser microstructure and greater fibrousness, which aligns with the SEM observations and further confirms that the extrudability of 20%WYP was superior to that of 20%YPP. The cause may be that the addition of yeast protein promoted protein molecule aggregation and induced the formation of a protein network structure with enhanced water-binding ability [35,36]. This effect may be attributed to the reduction in pore size between SPI and yeast protein, resulting in a denser structure. By enhancing the interactions between water and protein molecules, the whole yeast powder rendered the extrudates’ structure more compact. The improved structural integrity influenced the water distribution in the meat analogs, as a more compact structure led to reduced water loss during extrusion. The 20%WYP samples exhibited the most pronounced fibrous characteristics, and with texture data showing the highest fibrousness (1.84 ± 0.02, *p* < 0.05) at 20%WYP substitution. Moreover, greater water-holding capacity resulted in lower water mobility [37].

### 3.5. Intermolecular Interaction Analysis

Intermolecular interactions in the extrudates were assessed by evaluating changes in protein solubility across various selective extraction solutions [26,38].

**Figure 3:**
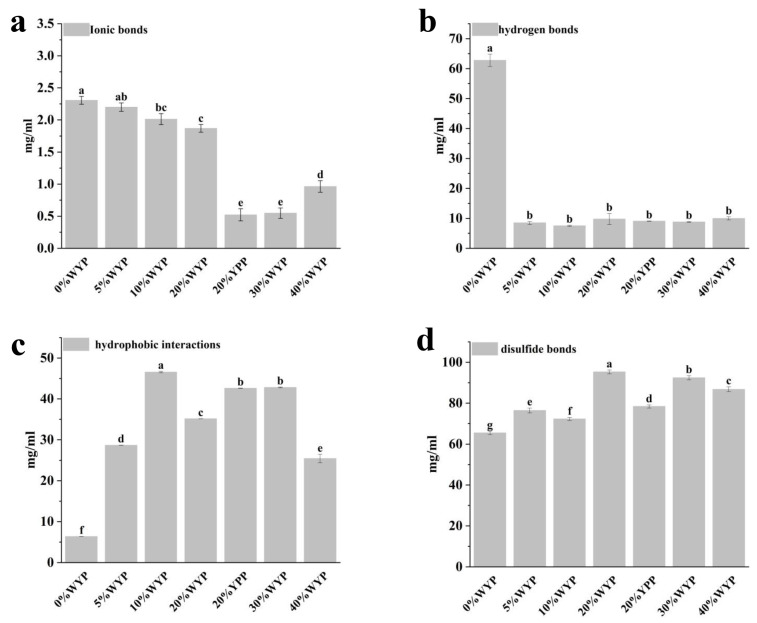
Comparison of intermolecular interactions in high-moisture mixed-protein extrudates across various substitution levels: (**a**) ionic interactions, (**b**) hydrogen bonding, (**c**) hydrophobic forces, and (**d**) disulfide linkages. Distinct lowercase letters denote statistically significant differences (*p* < 0.05).

As shown in Figure 3a, at low-to-moderate WYP substitution levels (5–20%), the ionic bond content gradually decreased with increasing WYP levels. At higher substitution levels (30–40%), further increases in WYP content led to a significant decline in ionic bond content. As shown in Figure 3b, the hydrogen bond content significantly decreased following the addition of WYP.

As shown in Figure 3c, after adding whole yeast powder, the hydrophobic interactions in all mixed-protein samples significantly increased. The cause may be that yeast protein itself contains a certain proportion of hydrophobic amino acids, which further contributes to enhancing hydrophobic interactions [39,40]. However, at 40%WYP substitution, hydrophobic interactions significantly decreased. This suggested an overall weakening of protein aggregation stability.

As shown in Figure 3d, after the addition of whole yeast powder, the disulfide bond content in the mixed-protein samples significantly increased, peaking at 20% WYP, consistent with the trend in Figure 3c. The increased disulfide bonds enhanced interactions between the two proteins, leading to tighter water binding at the microstructural level and resulting in fibrous aggregates [41]. The disulfide bond content of the 20%WYP sample (95.33 ± 0.92, *p* < 0.05) was higher than that of the 20%YPP sample (78.41 ± 0.78, *p* < 0.05), indicating that the extrudability of 20%WYP was superior to that of 20%YPP. The solubility of the extruded samples in different extraction solutions can be attributed to the disruption of specific intermolecular interactions. These results indicate that the overall structure of the protein extrudates is stabilized by a synergistic combination of multiple types of chemical interactions [42].

### 3.6. Surface Hydrophobicity Analysis

Hydrophobic interactions help maintain the protein structure and also affect the stability and functional properties of myofibrillar proteins. The surface hydrophobicity of the protein can be determined by the binding capacity of the hydrophobic chromophore bromophenol blue (BPB) [17]. In globular proteins, surface hydrophobicity reflects the extent of molecular unfolding, wherein previously buried nonpolar amino acid residues become exposed. An increase in surface hydrophobicity indicates the exposure of internal hydrophobic domains and potential denaturation of protein monomers [43].

**Figure 4:**
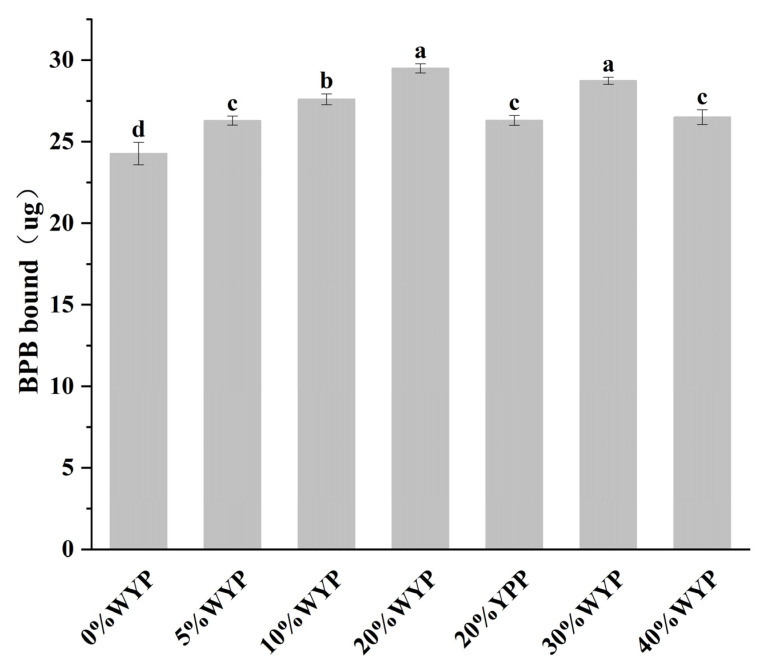
Surface hydrophobicity of high-moisture mixed-protein extrudates with varying whole yeast powder substitution levels. Different lowercase letters indicate statistically significant differences between samples (*p* < 0.05).

Figure 4 shows that as the whole yeast powder substitution level increased, the surface hydrophobicity of high-moisture mixed-protein extrudates significantly increased. This phenomenon is attributed to interactions between yeast protein and soybean protein, triggering protein structural reorganization and exposing hydrophobic groups initially buried within the structure [6]. Thus, whole yeast powder content positively correlated with the surface hydrophobicity of the samples. Specifically, as the substitution rate of whole yeast powder (WYP) increases from 5% to 40%, the surface hydrophobicity gradually increases. The 5% WYP, 20% YPP, and 40% WYP groups exhibit similar levels of hydrophobicity, while the 20% and 30% WYP groups reach a similar hydrophobicity threshold. Notably, the surface hydrophobicity peaked at a 20% WYP substitution, with a value of (29.51 ± 0.28, *p* < 0.05), significantly higher than that of the 20% YPP sample (26.31 ± 0.30, *p* < 0.05). These findings suggest that introducing whole yeast powder enhances network formation driven by hydrophobic interactions among proteins, demonstrating dose-dependent structural optimization and maximizing hydrophobic group exposure within a specific substitution range [44].

Both surface hydrophobicity and hydrophobic interactions were significantly enhanced at a moderate substitution level (20%WYP), indicating that the introduction of yeast protein promoted the exposure and aggregation of hydrophobic groups to some extent, thus facilitating protein rearrangement and fibrous structure formation during extrusion. However, excessive substitution (e.g., 40%WYP) increased surface hydrophobicity but significantly reduced hydrophobic interactions, possibly due to protein conformational instability or structural incompatibility, thereby hindering hydrophobic-driven protein network formation [10].

### 3.7. FTIR Analysis

The secondary structure of proteins in the extrudate samples was evaluated by analyzing the characteristic absorption peaks in the amide I region (1600–1700 cm^−1^) of the FTIR spectra. This region primarily originates from C=O stretching vibrations, accompanied by C–N stretching, C–N deformation, and N–H bending within the protein backbone. These vibrational modes provide both qualitative and quantitative insights into secondary structure elements, including α-helices, β-sheets, β-turns, and random coils [45].

**Figure 5:**
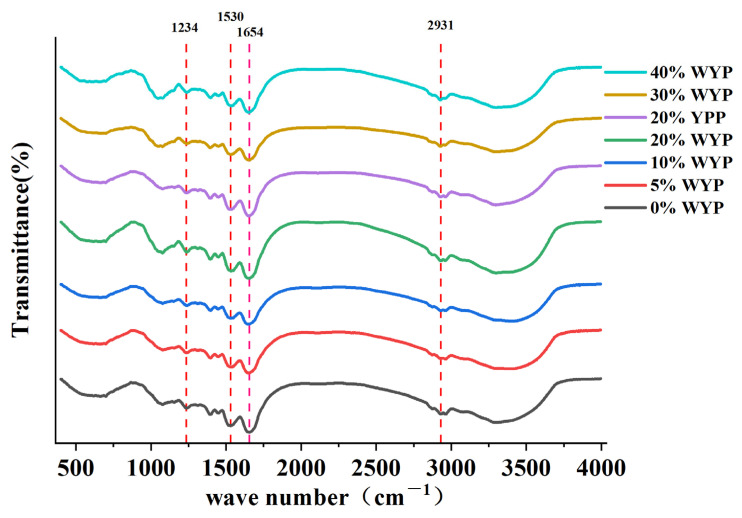
Fourier transform infrared (FTIR) spectra of meat analogs at different substitution levels.

As illustrated in Figure 5, the primary absorption peak of SPI was observed at 1654 cm^−1^, with additional peaks detected at 1530 cm^−1^ and 1234 cm^−1^, corresponding to C=O stretching, N–H bending, C–N stretching, and N–N bending vibrations, respectively. With the incorporation of whole yeast powder, increased damping of the C–H stretching vibrations in the 2800–3000 cm^−1^ region was observed, along with an intensified absorption peak at 1530 cm^−1^. This enhancement may be attributed to the introduction of additional C=O groups through the addition of whole yeast powder, resulting in stronger absorption in this region. A similar observation was reported by [46] when β-glucan was incorporated into SPI during high-moisture extrusion.

As presented in Table 4, at low-to-moderate substitution levels (0% WYP–20% WYP), the β-sheet content significantly increased (*p* < 0.05) with increasing whole yeast powder substitution, reaching its peak at 20% WYP (0.267 ± 0.003). Additionally, the β-sheet content of 20% WYP was significantly higher than that of 20% YPP (0.267 ± 0.003 > 0.260 ± 0.003, *p* < 0.05). At higher substitution levels (30% WYP–40% WYP), the β-sheet content tended to stabilize, remaining consistently higher than the control group (0% WYP). A similar increasing trend was also observed for antiparallel β-sheet structures, which reached their maximum at 20% WYP (0.123 ± 0.004, *p* < 0.05). The content of antiparallel β-sheets at 20% WYP was higher than that of 20% YPP (0.123 ± 0.004 > 0.119 ± 0.003, p < 0.05). This upward trend is consistent with the findings reported by [47], who suggested that the formation of antiparallel β-sheets may be associated with the development of fibrous structures in extruded protein systems. Overall, the β-sheet content exhibited an increasing trend (*p* < 0.05) with rising WYP substitution levels, consistent with previous reports on structural transitions in extruded protein systems [10]. In contrast, no notable changes (*p* > 0.05) were observed in α-helix content across the different substitution levels, which is also in agreement with earlier findings [10], indicating that the dominant conformational shift occurred primarily in the β-sheet domains. The increase in β-sheet structures at low-to-moderate substitution levels contributed to fibrous protein structure formation and strengthened protein–protein interactions. These findings align with [48], who noted that increased β-sheet content facilitated fiber formation. The growth in β-sheet structures is closely associated with protein aggregation. During high-moisture extrusion, proteins undergo deformation and aggregate, ultimately forming aligned fibrous structures in plant-based meat analogues [49]. This structural transition indicates that β-sheet formation may be the key driver of protein alignment and anisotropic texture development in high-moisture extruded systems.

## 4. Conclusions

This study investigated the effects of incorporating whole yeast powder (WYP) into soy protein on the structural and textural characteristics of high-moisture extruded meat analogs. Compared with yeast protein powder (YPP), WYP substitution improved fibrous structure and protein network stability within a moderate substitution range. Appropriate levels of WYP enhanced fiber alignment and water-holding capacity, resulting in a denser internal structure. At higher levels, the fibrous characteristics declined, indicating a concentration-dependent effect. While YPP has higher protein purity, WYP provides additional components such as dietary fiber and lipids, which may promote protein aggregation and alignment under high-moisture, high-shear conditions. The addition of WYP improved quality attributes, including hardness, chewiness, and fibrous integrity. These findings support the use of WYP as a functionally effective and economically viable alternative to YPP in plant-based meat formulations. Moderate inclusion of WYP enhances fibrous structure formation and texture in soy-based meat analogs. The results provide technical guidance for optimizing protein blends and improving extrudability in industrial applications.

The interaction mechanisms among proteins under high-moisture extrusion conditions are complex and multifactorial. Given the promising application potential of whole yeast powder (WYP) in meat analogue production, further systematic investigation into its regulatory effects on plant protein interactions during high-moisture extrusion is warranted. Moreover, as a novel food-grade protein resource, the physicochemical properties and functional functionalities of WYP require comprehensive characterization.

## Figures and Tables

**Table 1:** The proportion of dry protein content in different raw material samples.

Sample Number	WYP%	SPI%	YPP%
0% WYP	0	100	0
5% WYP	5	95	0
10% WYP	10	90	0
20% WYP	20	80	0
20% YPP	0	80	20
30% WYP	30	70	0
40% WYP	40	60	0

**Table 2:** Color changes of high-moisture mixed-protein extrudates at different substitution levels.

	L*	a*	b*	*ΔE*
0%WYP	37.89 ± 0.26 ^e^	3.17 ± 0.14 ^b^	14.86 ± 0.45 ^c^	0.00
5%WYP	37.40 ± 0.06 ^e^	3.17 ± 0.21 ^b^	15.37 ± 0.62 ^c^	0.71 ± 0.03 ^e^
10%WYP	41.11 ± 0.24 ^d^	3.19 ± 0.96 ^b^	14.67 ± 0.09 ^c^	3.23 ± 0.07 ^d^
20%WYP	47.00 ± 0.45 ^b^	2.65 ± 0.81 ^c^	16.39 ± 0.28 ^b^	9.24 ± 0.06 ^b^
20%YPP	44.95 ± 0.38 ^c^	3.20 ± 0.04 ^b^	16.35 ± 0.37 ^b^	7.22 ± 0.30 ^c^
30%WYP	47.47 ± 0.55 ^b^	3.85 ± 0.21 ^a^	17.57 ± 0.29 ^a^	9.04 ± 0.11 ^b^
40%WYP	49.16 ± 0.70 ^a^	3.91 ± 0.04 ^a^	18.22 ± 0.02 ^a^	11.80 ± 0.24 ^a^

Data were presented as mean ± standard deviation (SD) for three replicates (n = 3). Differences among means were evaluated using Tukey’s HSD (Honestly Significant Difference) test with a significance threshold of *p* < 0.05. Values in the same column marked with different lowercase letters indicate statistically significant differences.

**Table 3:** Texture analysis of high-moisture mixed-protein extrudates at various substitution levels.

	Fibrousness (Perpendicular Hardness/Parallel Hardness)	Hardness (N)		Chewiness (N)	
		Parallel (FL)	Perpendicular (FV)	Parallel	Perpendicular
0%WYP	1.44 ± 0.02 ^d^	494.31 ± 11.49 ^d^	698.92 ± 20.18 ^e^	653.35 ± 21.76 ^c^	686.44 ± 20.41 ^d^
5%WYP	1.45 ± 0.02 ^cd^	522.83 ± 10.40 ^cd^	760.51 ± 16.96 ^d^	648.66 ± 15.86 ^c^	686.21 ± 11.35 ^d^
10%WYP	1.56 ± 0.04 ^c^	561.94 ± 16.37 ^bc^	879.23 ± 15.27 ^c^	674.74 ± 8.15 ^bc^	705.91 ± 15.54 ^cd^
20%WYP	1.84 ± 0.02 ^a^	574.93 ± 5.84 ^b^	1054.84 ± 1 6.91 ^a^	709.51 ± 9.17 ^abc^	736.12 ± 21.55 ^bc^
20%YPP	1.81 ± 0.04 ^ab^	531.18 ± 17.34 ^cd^	959.53 ± 25.92 ^b^	653.34 ± 40.12 ^c^	685.16 ± 20.72 ^d^
30%WYP	1.73 ± 0.05 ^b^	582.78 ± 32.73 ^b^	998.82 ± 10.22 ^b^	728.30 ± 17.21 ^ab^	765.10 ± 11.47 ^ab^
40%WYP	1.69 ± 0.06 ^b^	627.04 ± 9.58 ^a^	1062.04 ± 6.60 ^a^	740.75 ± 27.56 ^a^	795.61 ± 12.59 ^a^

Each value represents the mean ± standard deviation (n = 3). Statistically significant differences (*p* < 0.05) among means in the same column are indicated by different lowercase letters, as determined by Tukey’s HSD test.

**Table 4:** Changes in the secondary structure of high-moisture blended protein extrudates at different substitution levels.

	β-Sheet	Random Coil	α-Helix	β-Turn	β-Antiparallel
0%WYP	0.254 ± 0.001 ^e^	0.224 ± 0.002 ^ab^	0.219 ± 0.006 ^a^	0.191 ± 0.002 ^a^	0.112 ± 0.002 ^d^
5%WYP	0.257 ± 0.002 ^d^	0.230 ± 0.006 ^a^	0.225 ± 0.003 ^a^	0.178 ± 0.003 ^b^	0.116 ± 0.001 ^bc^
10%WYP	0.263 ± 0.001 ^bc^	0.227 ± 0.001 ^ab^	0.224 ± 0.004 ^a^	0.169 ± 0.001 ^b^	0.118 ± 0.001 ^bc^
20%WYP	0.267 ± 0.003 ^a^	0.229 ± 0.001 ^ab^	0.219 ± 0.003 ^a^	0.167 ± 0.002 ^b^	0.123 ± 0.004 ^a^
20%YPP	0.260 ± 0.003 ^c^	0.226 ± 0.002 ^ab^	0.222 ± 0.001 ^a^	0.172 ± 0.002 ^b^	0.119 ± 0.003 ^b^
30%WYP	0.266 ± 0.002 ^ab^	0.226 ± 0.001 ^b^	0.223 ± 0.001 ^a^	0.169 ± 0.004 ^c^	0.115 ± 0.003 ^cd^
40%WYP	0.265 ± 0.001 ^ab^	0.227 ± 0.002 ^ab^	0.221 ± 0.001 ^a^	0.171 ± 0.002 ^c^	0.115 ± 0.004 ^cd^

Means labeled with different lowercase letters in the same column differ significantly based on Tukey’s HSD test (*p* < 0.05).

## Data Availability

The original contributions presented in the study are included in the article, further inquiries can be directed to the corresponding author.

## Supplementary Materials

The following supporting information can be downloaded at: https://www.mdpi.com/article/10.3390/foods14142479/s1, Figure S1: Front view of twin-screw extruder; Figure S2: Cooling mould schematic; Figure S3: Fourier self-deconvolution curve fitting spectra of extrudates with different whole yeast powder additions; Figure S4: Orientation distribution analysis of extruded samples with different levels of whole yeast powder (WYP) or yeast protein (YPP) substitution. The fiber orientation was quantified using Orientation based on SEM images (×1500).
