# Supplementary material for: Development of Soy-Based Meat Analogues via Wet Twin-Screw Extrusion: Enhancing Textural and Structural Properties Through Whole Yeast Powder Supplementation
Source: Foods. 2025 Jul 15;14(14):2479. doi: 10.3390/foods14142479 (PMC12294595; doi:10.3390/foods14142479)
Supplement: Supplementary file 1 [file foods-14-02479-s001.zip › Supplementary Materials.pdf]

# Supplementary information

**Title:** Development of Soy-Based Meat Analogues via Wet Twin-Screw Extrusion: Enhancing Textural and Structural Properties through Whole Yeast Powder Supplementation

**Authors:** *Shikang Tang*[1,2,3,4], *Yidian Li*[1,2,3,4], *Xuejiao Wang*[1,2,3,4], *Linyan Zhou*[1,2,3,4], *Zhijia Liu*[1,2,3,4], *Lianzhou Jiang*[5], *Chaofan Guo*[1,2,3,4*], *Junjie Yi*[1,2,3,4]

1 Faculty of Food Science and Engineering, Kunming University of Science and Technology, Kunming 650500, China

15969040899@163.com (S.T.); liyidian0901@163.com (Y.L.); wangxuejiao173@hotmail.com (X.W.);

zhoulinyan916@hotmail.com (L.Z); zhijia_liu@outlook.com (Z.L.); junjieyi@kust.edu.cn (J.Y.)

2 Key Laboratory of Plateau Characteristic Prepared Food in Yunnan Province, Kunming 650500, China

3 Yunnan International Joint Laboratory of Green Food Processing, Kunming 650500, China

4 International Green Food Processing Research and Development Center of Kunming City, Kunming 650500, China

5 College of Food Science, Northeast Agricultural University, Harbin, 150030, China

jlzname@163.com (L.J.)

*Correspondence: guochaofanfan@outlook.com Tel.: 15606183166

**Table of Content:**

**Figures:**

**Figure. S1.** Front view of twin-screw extruder

**Figure. S2.** Cooling mould schematic

**Figure. S3.** Fourier self-deconvolution curve fitting spectra of extrudates with different whole yeast powder additions：（a）0%WYP，（b）5%WYP, (c) 10%WYP，（d） 20%WYP, （e）20%YPP, (f) 30%WYP，（g）40%WYP.

**Figure. S4.** Orientation distribution analysis of extruded samples with different levels of whole yeast powder (WYP) or yeast protein (YPP) substitution. The fiber orientation was quantified using Orientation based on SEM images (×1500)：（a）0%WYP，(b) 5%WYP, (c) 10%WYP，(d) 20%WYP，(e) 20%YPP，(f) 30%WYP，(g) 40%WYP.

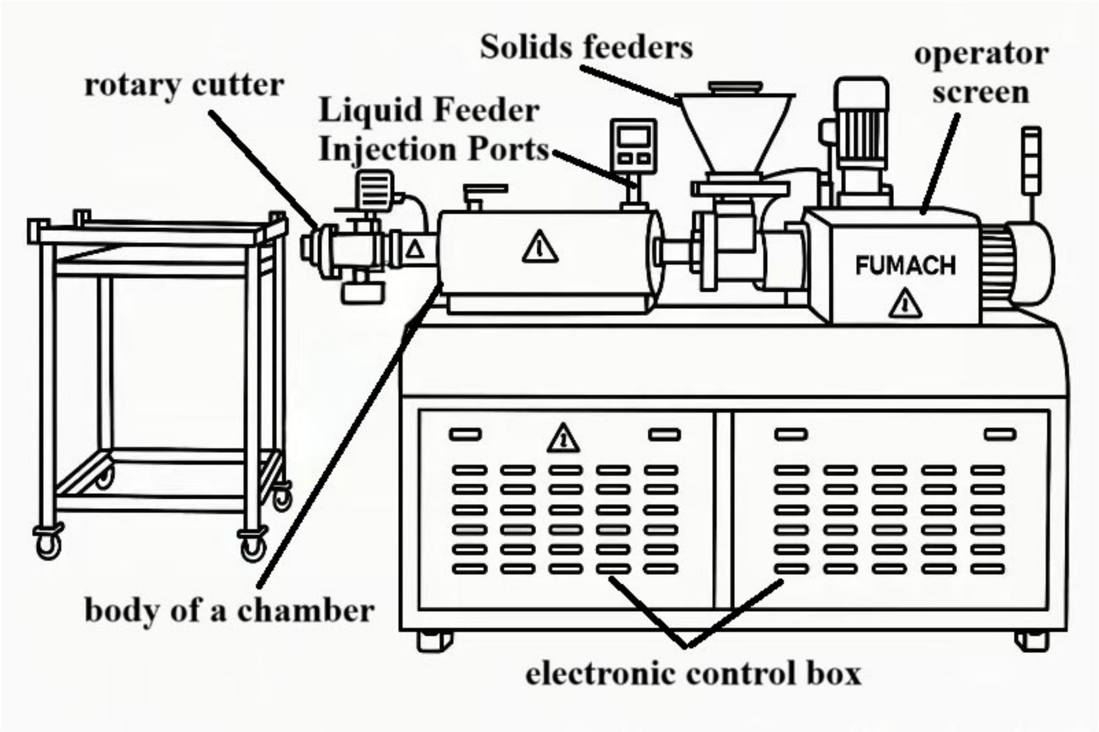


**Figure. S1.** Front view of twin-screw extruder

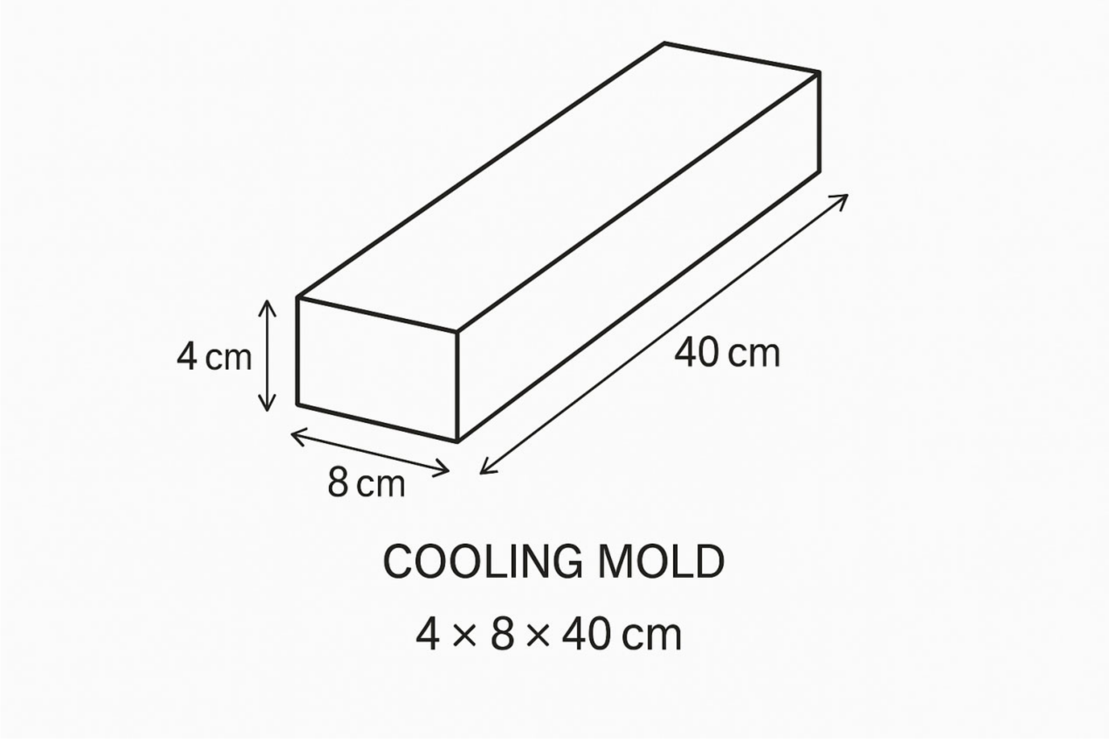


**Figure. S2.** Cooling mould schematic

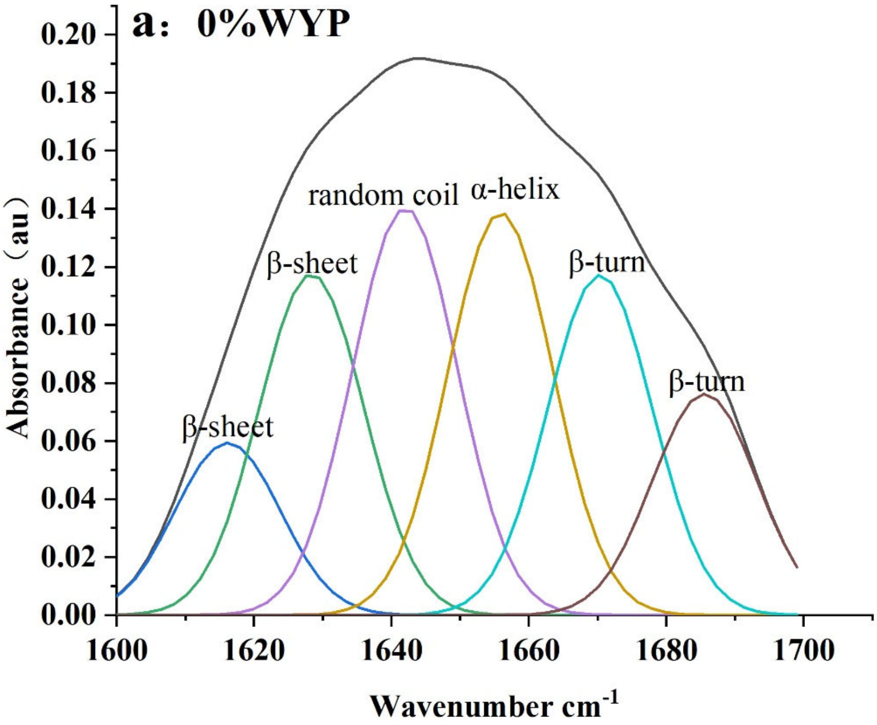
a：0%WYP
random coil
α-helix
β-sheet
β-turn
β-turn
β-sheet
Absorbance（au）
Wavenumber cm-1
0.20
0.18
0.16
0.14
0.12
0.10
0.08
0.06
0.04
0.02
0.00
1600
1620
1640
1660
1680
1700

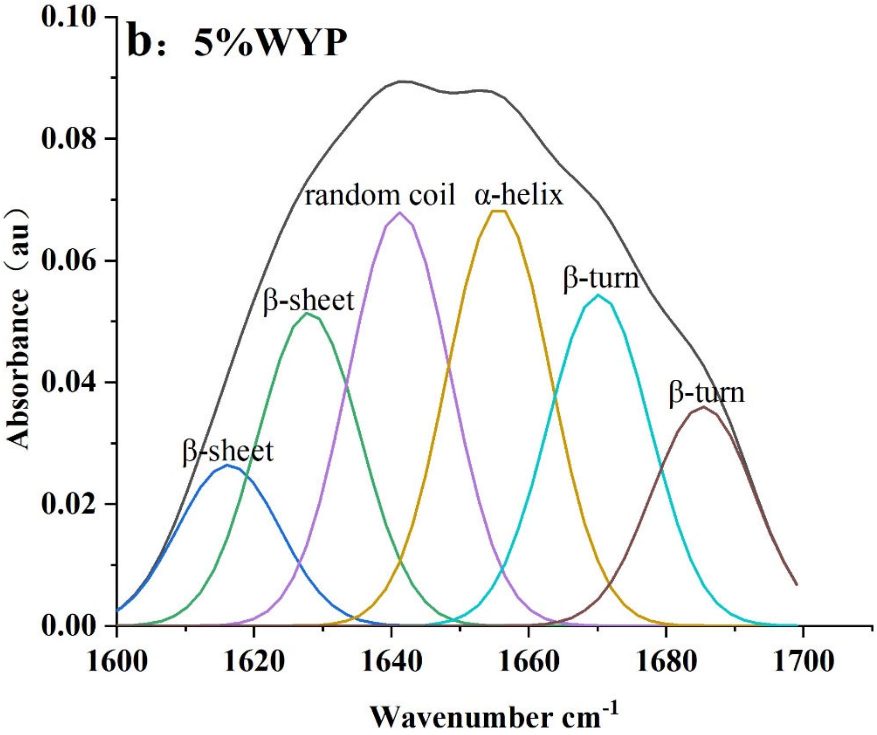
b：5%WYP
random coil
α-helix
β-sheet
β-turn
β-turn
β-sheet
Absorbance（au）
Wavenumber cm-1
0.10
0.08
0.06
0.04
0.02
0.00
1600
1620
1640
1660
1680
1700

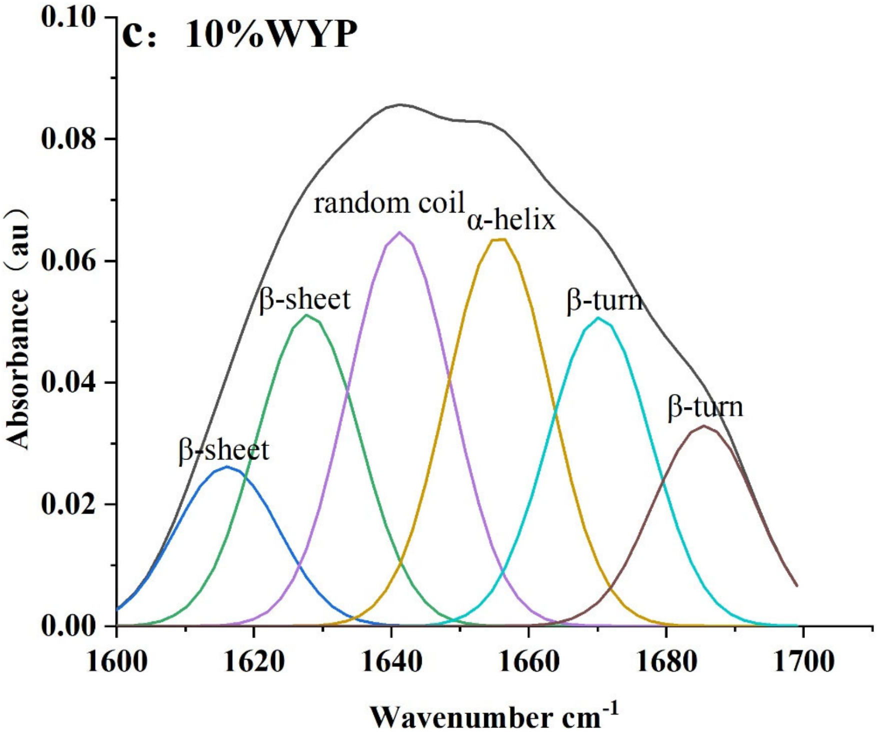
c：10%WYP
random coil
α-helix
β-sheet
β-turn
β-turn
β-sheet
Absorbance（au）
0.10
0.08
0.06
0.04
0.02
0.00
1600
1620
1640
1660
1680
1700
Wavenumber cm-1

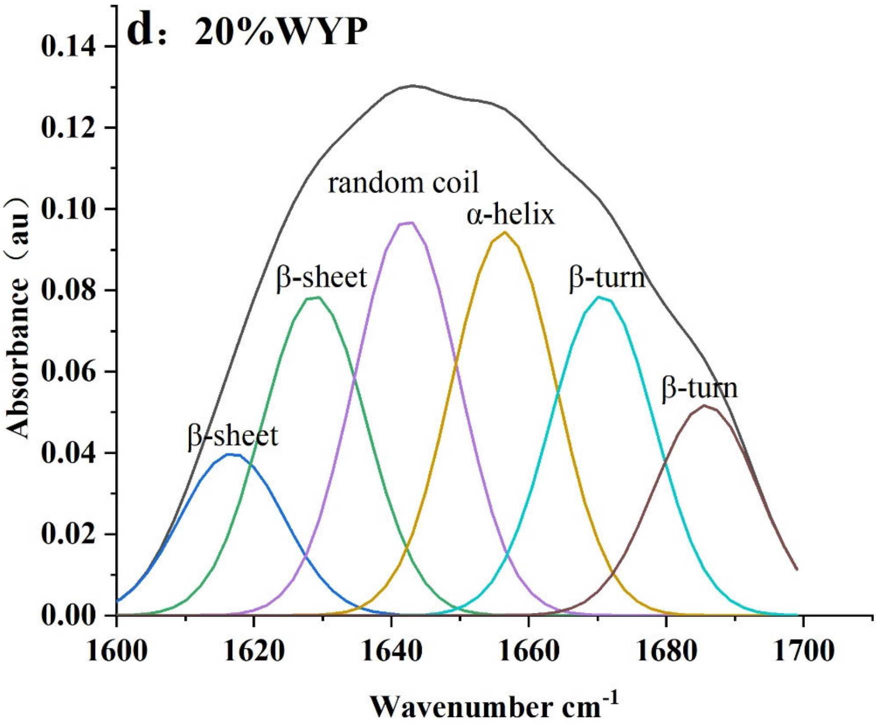
d：20%WYP
random coil
α-helix
β-sheet
β-turn
β-turn
β-sheet
Absorbance（au）
0.14
0.12
0.10
0.08
0.06
0.04
0.02
0.00
1600
1620
1640
1660
1680
1700
Wavenumber cm-1

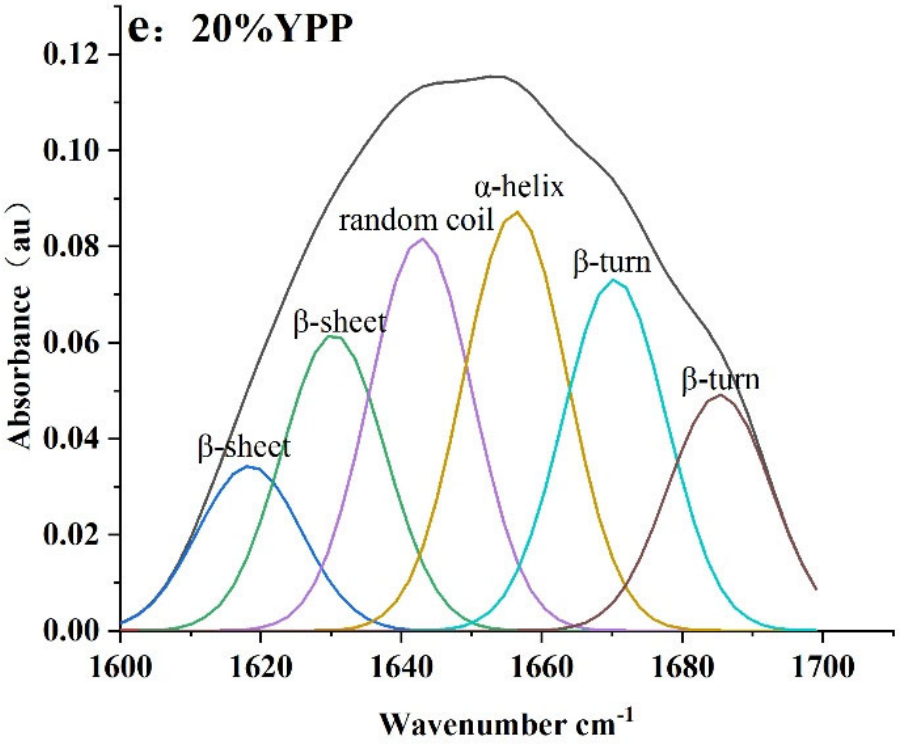
e：20%YPP
0.12
0.10
0.08
0.06
0.04
0.02
0.00
Absorbance（au）
1600
1620
1640
1660
1680
1700
Wavenumber cm-1
β-sheet
β-sheet
random coil
α-helix
β-turn
β-turn

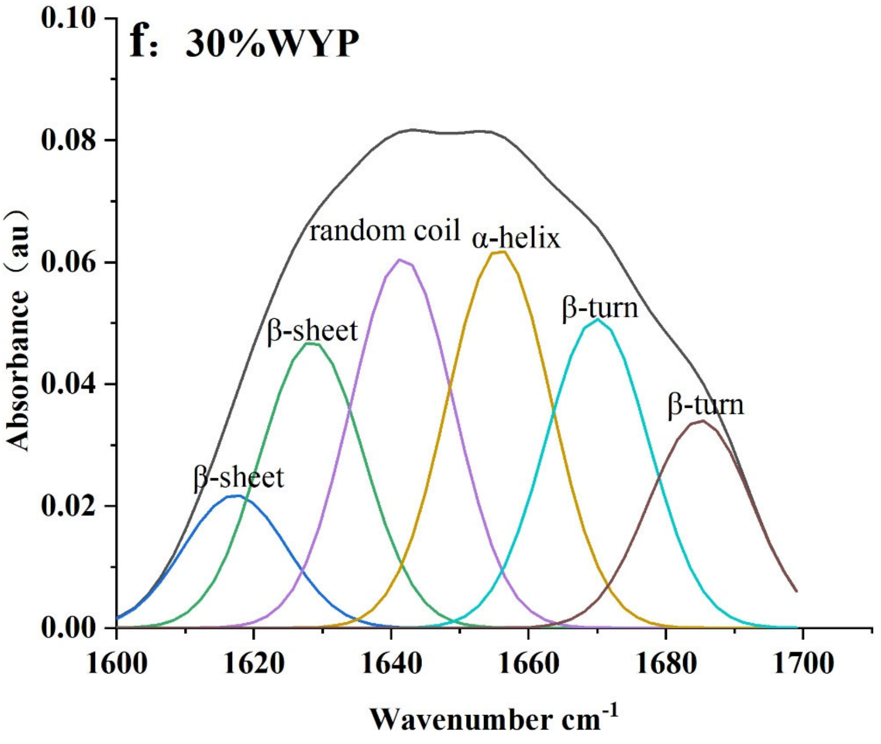
f：30%WYP
0.10
0.08
0.06
0.04
0.02
0.00
Absorbance（au）
1600
1620
1640
1660
1680
1700
Wavenumber cm-1
β-sheet
β-sheet
random coil
α-helix
β-turn
β-turn

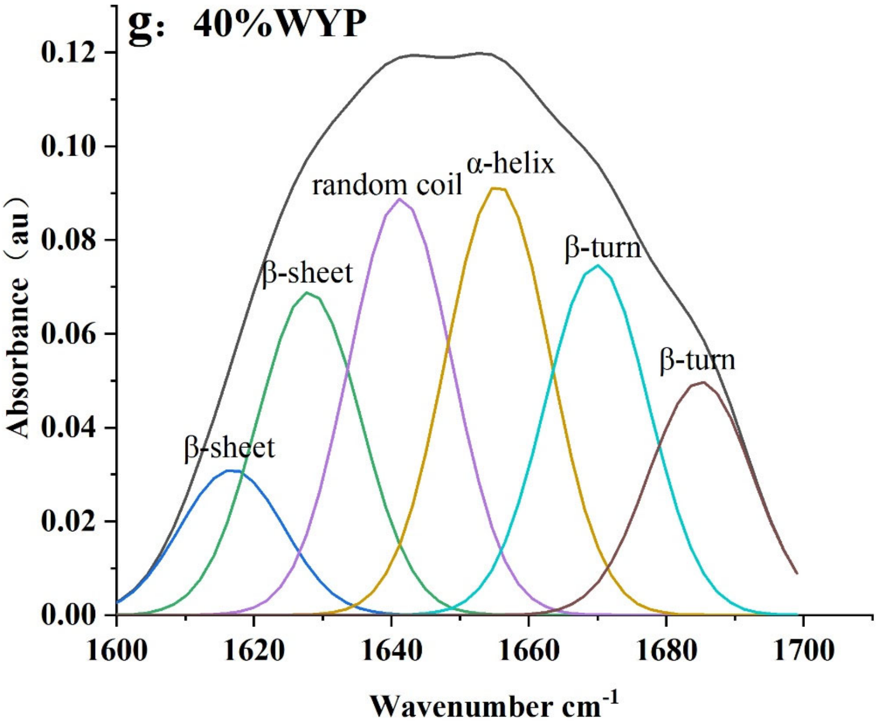


**Figure. S3.** Fourier self-deconvolution curve fitting spectra of extrudates with different whole yeast powder additions：（a）0%WYP，（b）5%WYP, (c) 10%WYP，（d）20%WYP, （e）20%YPP, (f) 30%WYP，（g）40%WYP.

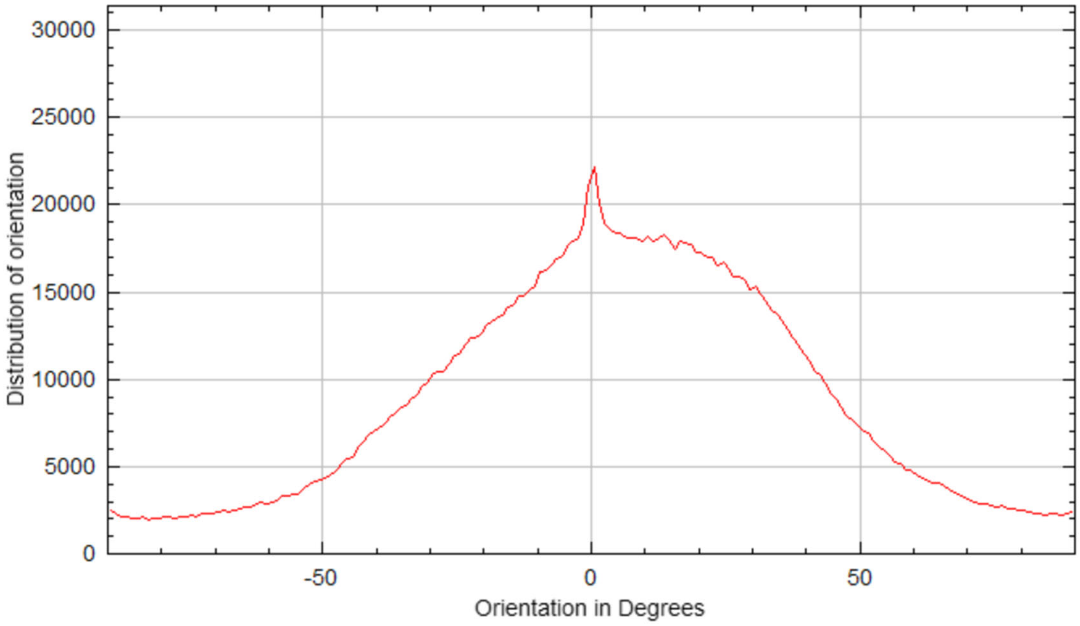


（a）0%WYP

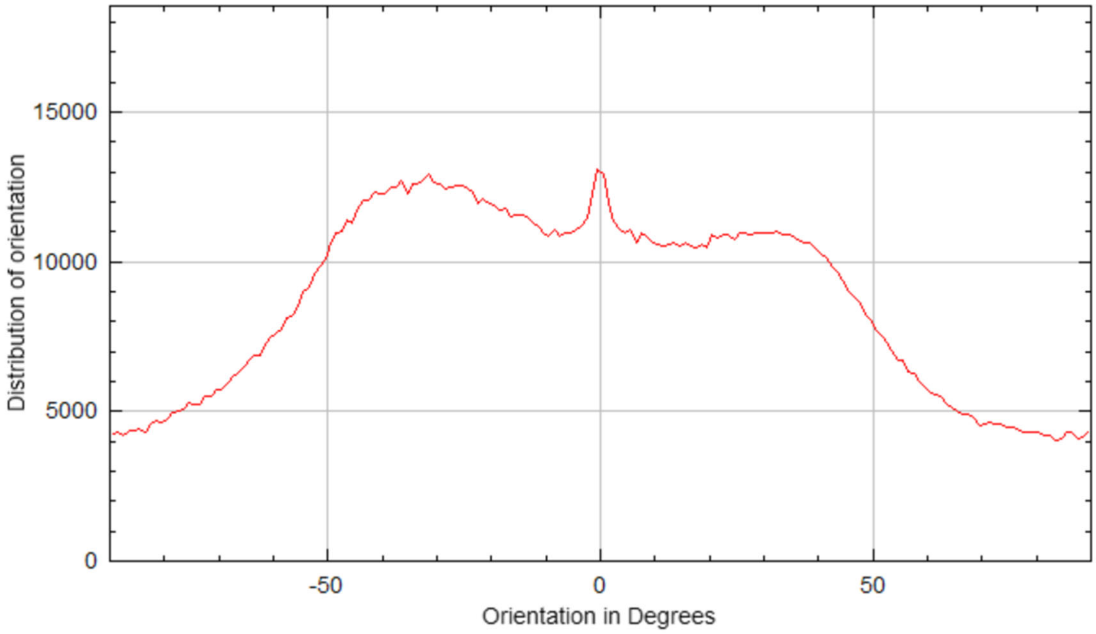


（b）5%WYP

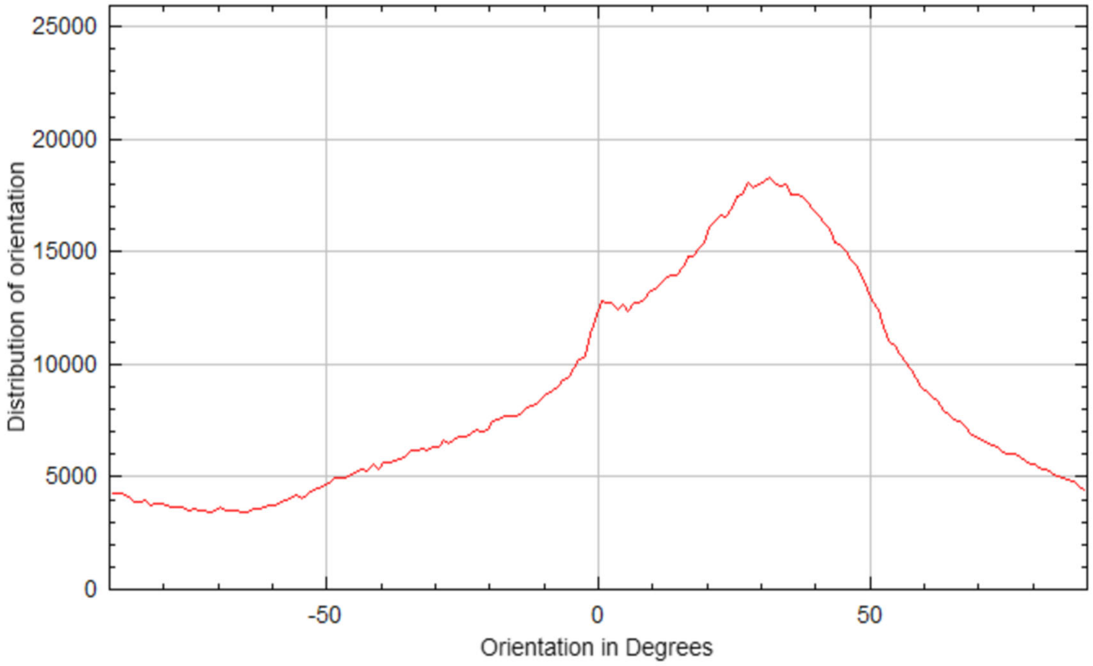


（c）10%WYP

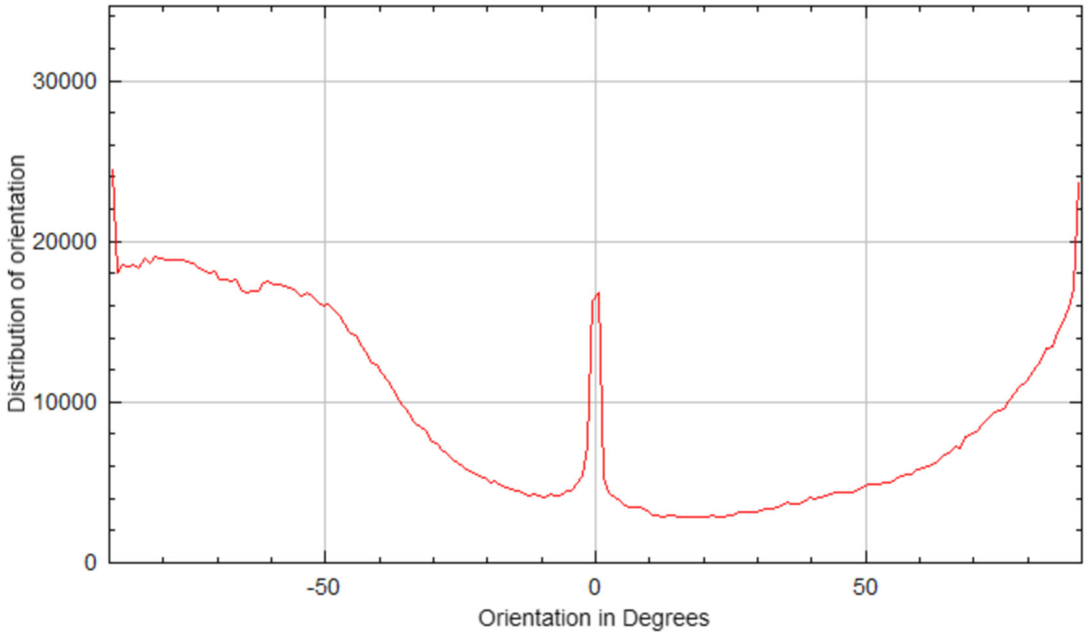


（d）20%WYP

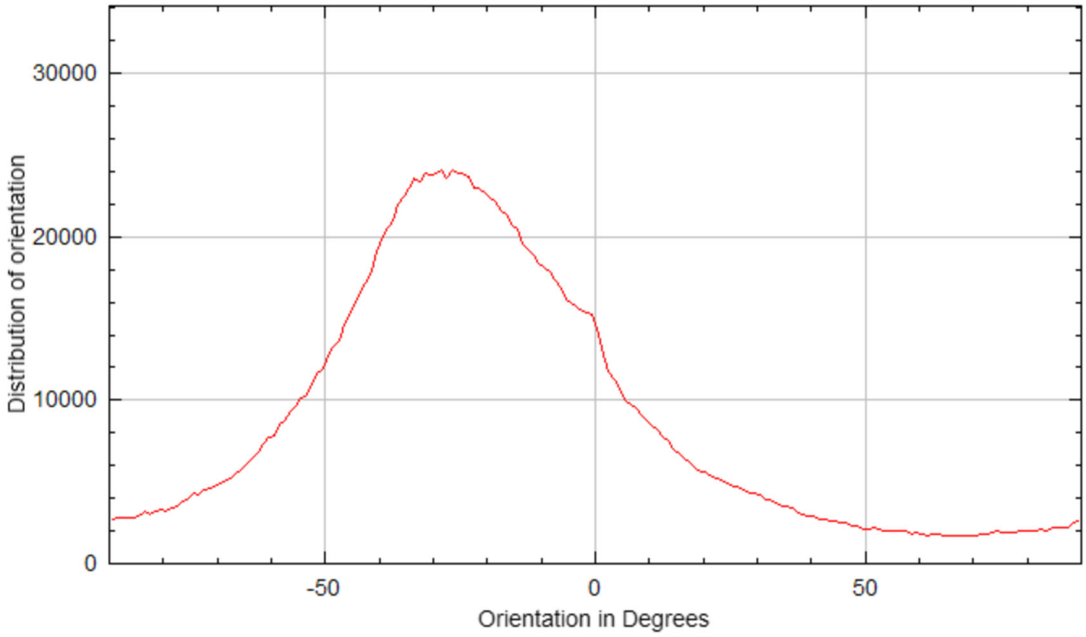


（e）20%YPP

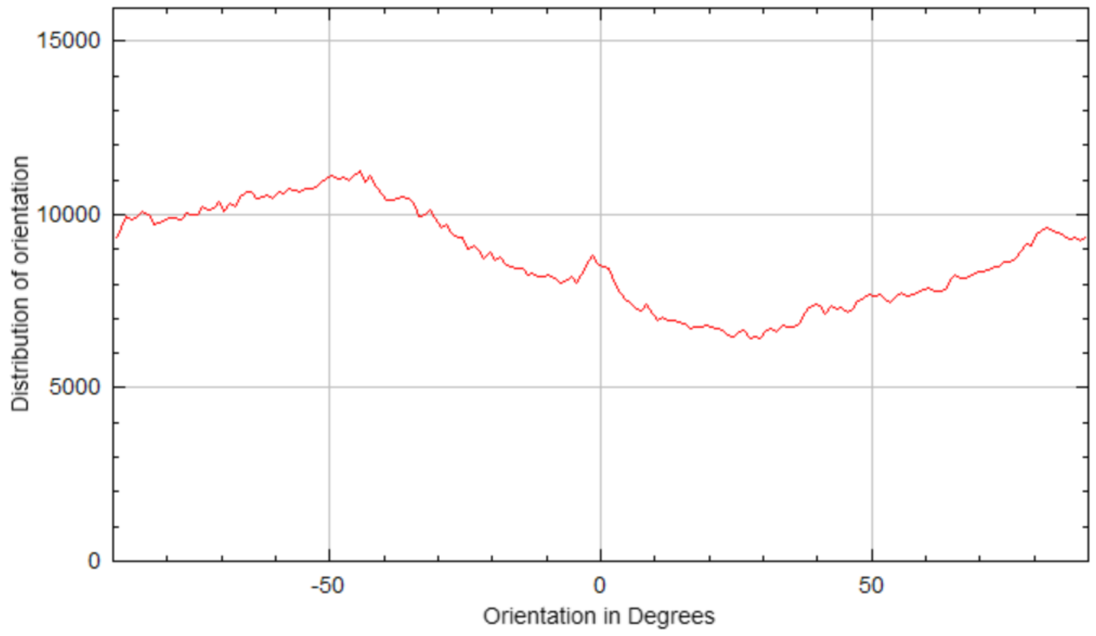


（f）30%WYP

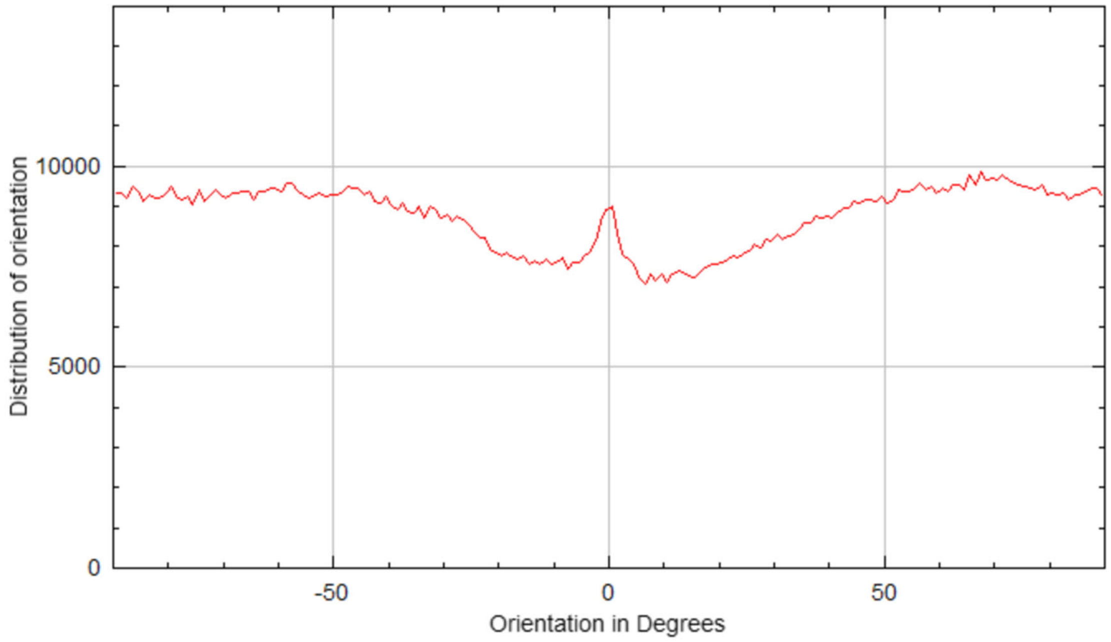


（g）40%WYP

**Figure. S4.** Orientation distribution analysis of extruded samples with different levels of whole yeast powder (WYP) or yeast protein (YPP) substitution. The fiber orientation was quantified using Orientation based on SEM images (×1500)：（a）0%WYP，（b）5%WYP, (c) 10%WYP，（d）20%WYP,（e）20%YPP, (f) 30%WYP，（g）40%WYP.
